# Whole‐cell biocatalytic and de novo production of alkanes from free fatty acids in *Saccharomyces cerevisiae*

**DOI:** 10.1002/bit.25920

**Published:** 2016-01-19

**Authors:** Jee Loon Foo, Adelia Vicanatalita Susanto, Jay D. Keasling, Susanna Su Jan Leong, Matthew Wook Chang

**Affiliations:** ^1^ Department of Biochemistry, Yong Loo Lin School of MedicineNational University of SingaporeSingapore; ^2^ NUS Synthetic Biology for Clinical and Technological Innovation (SynCTI), Life Sciences InstituteNational University of SingaporeSingapore; ^3^ Joint BioEnergy InstituteEmeryvilleCalifornia; ^4^ Biological Systems and Engineering DivisionLawrence Berkeley National LaboratoryBerkeleyCalifornia; ^5^ Department of Chemical and Biomolecular Engineering and Department of BioengineeringUniversity of CaliforniaBerkeleyCalifornia; ^6^ Singapore Institute of TechnologySingapore

**Keywords:** alkane, aldehyde, fatty acid, biofuels, metabolic engineering, whole‐cell biocatalysis, de novo biosynthesis

## Abstract

Rapid global industrialization in the past decades has led to extensive utilization of fossil fuels, which resulted in pressing environmental problems due to excessive carbon emission. This prompted increasing interest in developing advanced biofuels with higher energy density to substitute fossil fuels and bio‐alkane has gained attention as an ideal drop‐in fuel candidate. Production of alkanes in bacteria has been widely studied but studies on the utilization of the robust yeast host, *Saccharomyces cerevisiae*, for alkane biosynthesis have been lacking. In this proof‐of‐principle study, we present the unprecedented engineering of *S. cerevisiae* for conversion of free fatty acids to alkanes. A fatty acid α‐dioxygenase from *Oryza sativa* (rice) was expressed in *S. cerevisiae* to transform C_12–18_ free fatty acids to C_11–17_ aldehydes. Co‐expression of a cyanobacterial aldehyde deformylating oxygenase converted the aldehydes to the desired alkanes. We demonstrated the versatility of the pathway by performing whole‐cell biocatalytic conversion of exogenous free fatty acid feedstocks into alkanes as well as introducing the pathway into a free fatty acid overproducer for de novo production of alkanes from simple sugar. The results from this work are anticipated to advance the development of yeast hosts for alkane production. Biotechnol. Bioeng. 2017;114: 232–237. © 2016 The Authors. *Biotechnology and Bioengineering* Published by Wiley Periodicals, Inc.

In view of concerns regarding depletion of petroleum resources and environmental issues arising from heavy reliance on fossil fuel for energy, development of microbial biosynthesis of advanced biofuels for generating sustainable and renewable energy sources has gathered much attention. Alkane is an ideal biofuel candidate because it is a major component of fossil fuel and is high in energy density (Lee et al., 2008; Rude and Schirmer, 2009). In the past few years, production of alkane in bacterial hosts has been extensively explored (Choi and Lee, 2013; Schirmer et al., 2010) but research on alkane biosynthesis in yeast lags far behind. The yeast *Saccharomyces cerevisiae* has been used extensively for commercial fermentation because it has several advantages over bacterial systems, such as higher tolerance to harsh fermentation conditions and insusceptibility to phage contamination (Hong and Nielsen, 2012). Despite *S. cerevisiae* being a robust industrial yeast production host, production of alkanes in *S. cerevisiae* was reported only recently. To date, the alkane biosynthetic pathways implemented in *S. cerevisiae* involve the conversion of fatty acyl‐CoAs to aldehydes using fatty acyl‐CoA reductases (FARs) and subsequent biotransformation of the aldehyde intermediates to alkanes by plant aldehyde decarbonylase or cyanobacterial aldehyde deformylating oxygenase (cADO) (Bernard et al., 2012; Buijs et al., 2014). However, the aldehyde production was limited and not directly detectable in these pathways, thus contributing to the low production titer of alkanes.

Herein, we report a proof‐of‐principle study on the engineering of *S. cerevisiae* for improved whole‐cell biocatalytic and de novo production of aldehydes, and medium‐ and long‐chain alkane biosynthesis using free fatty acid (FFA) precursors (Fig. 1). The use of FFAs as precursors instead of fatty acyl‐CoAs in the heterologous alkane production pathway has two main advantages. First, unlike FAR‐dependent pathways, exogenous FFA feedstocks can be used directly as substrates in our proposed pathway for whole‐cell biocatalysis to produce alkanes without the need for an additional enzymatic step to biosynthesize fatty acyl‐CoA. Second, de novo overproduction of FFAs from fermentable sugar is more easily achieved than overproduction of fatty acyl‐CoAs because biosynthesis of the latter is tightly regulated by feedback inhibition (Runguphan and Keasling, 2014). Therefore, implementation of our alkane production pathway in an FFA‐overproducing *S. cerevisiae* strain for de novo alkane biosynthesis could have a greater potential than using an FAR‐dependent pathway for high level production of aldehyde intermediates and, consequently, high alkane titers.

**Figure 1:**
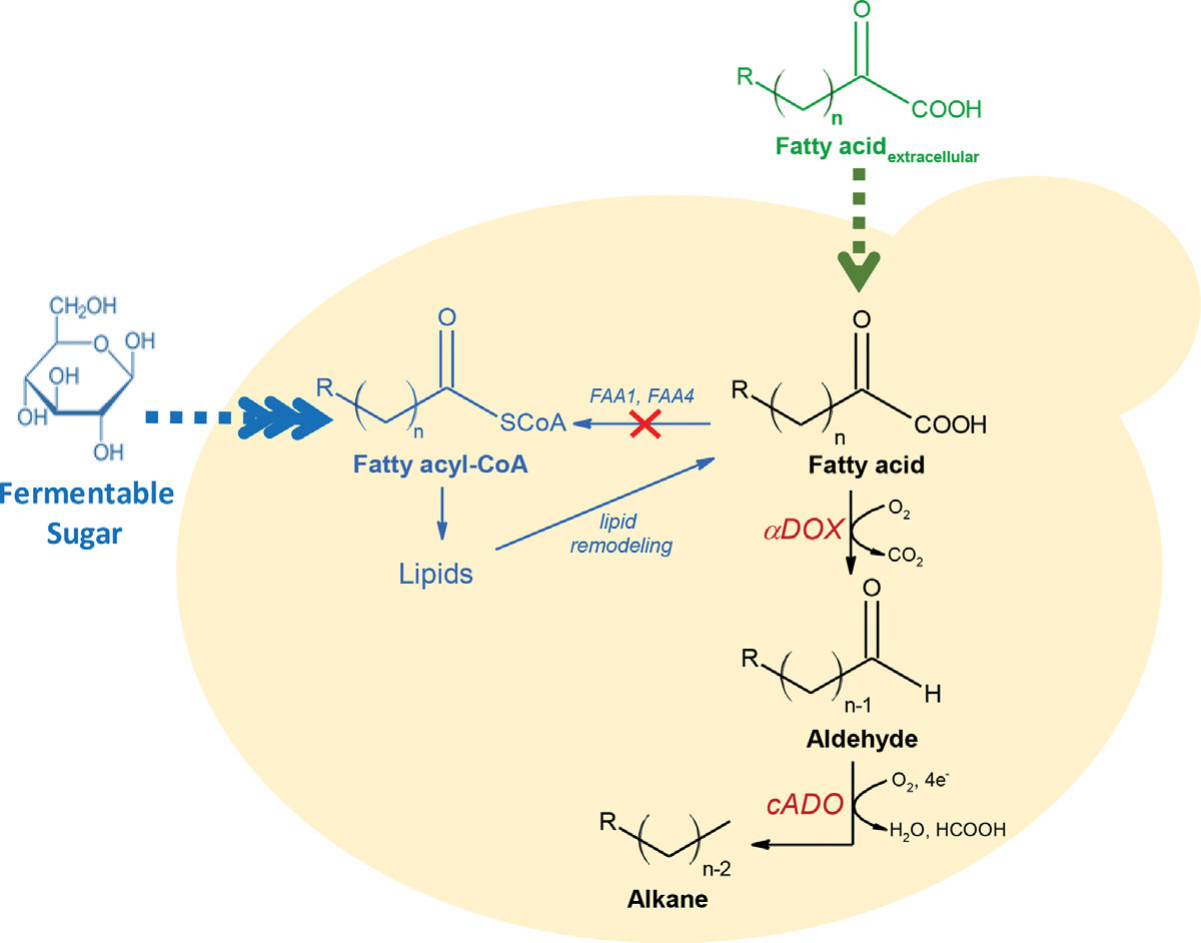
Schematic diagram for alkane production in *S. cerevisiae* from free fatty acids. An alkane production pathway that utilized a fatty acid α‐dioxygenase (αDOX) for conversion of free fatty acids to aldehydes and subsequent production of alkanes by deformylation of the aldehydes with cyanobacterial aldehyde deformylating oxygenase (cADO) was introduced into *S. cerevisiae*. The resulting strain can function as a whole‐cell biocatalyst to produce alkane from extracellular fatty acid sources (green path). By deleting *FAA1* and *FAA4* in *S. cerevisiae* to allow accumulation of free fatty acid precursors derived from intracellular lipid remodeling and subsequent introduction of the alkane production pathway, a yeast strain for de novo production of alkane from fermentable sugar was developed (blue path).

The biochemical pathway for aldehyde and alkane production in this study is illustrated in Figure 1. We utilized a fatty acid α‐dioxygenase (αDOX) from *Oryza sativa* (rice) (Koeduka et al., 2002) for production of aldehyde from FFAs in *S. cerevisiae*. This enzyme hydroperoxidates the α‐carbon of fatty acids and the 2‐hydroperoxide intermediate products spontaneously decarboxylate into C_n−1_ aldehydes. To produce alkanes, we co‐expressed cADO from *Synechococcus elongatus* PCC 7942 (Schirmer et al., 2010) to deformylate the aldehyde precursors to C_n−2_ alkanes. The advantage of utilizing αDOX in the pathway is that, unlike FARs, αDOX uses dioxygen instead of NADPH for production of aldehyde (Koeduka et al., 2002). Therefore, aldehyde production with αDOX will not compete with essential metabolic pathways, such as fatty acid synthesis pathway, for the limited NADPH pool or be easily compromised due to insufficient co‐factors since dioxygen is abundant in the air.

To develop our alkane biosynthesis pathway in *S. cerevisiae*, we first optimized the whole‐cell biocatalytic production of aldehydes and alkanes. An αDOX‐expressing *S. cerevisiae* strain JL1 was generated by integrating the gene encoding αDOX into the genome and overexpressing it under the strong constitutive TEF1 promoter. In the absence of exogenous FFAs, JL1‐ctrl (i.e., JL1 with an empty pYES2/CT plasmid) cultivated in defined media produced only miniscule amounts of pentadecanal (18.2 μg/L) due to the low level of endogenous FFAs in wild‐type *S. cerevisiae* (Runguphan and Keasling, 2014). Upon supplementing the culture medium with 200 mg/L even chain‐length C_12–18_ free saturated fatty acids, low levels of the corresponding C_n−1_ odd chain‐length C_11–17_ aldehydes were produced (<40 μg/L) (Fig. 2A). Concurrently, 1‐undecanol and 1‐tridecanol were detected, indicating that endogenous alcohol dehydrogenases reduce the medium chain aldehydes to alcohols. We hypothesized that the low aldehyde titer was due to the low solubility of FFAs limiting their uptake for conversion to aldehyde. Therefore, we raised the pH of the growth medium to pH 5.8 and 7.0 with phosphate buffer to increase FFA solubility; higher pHs were not investigated because alkaline pH inhibits growth (Pena et al., 2015). Consequently, biotransformation of all chain lengths of FFAs to aldehydes was markedly enhanced with increasing pH. At pH 5.8, there was more undecanal produced than the longer chain‐length aldehydes, possibly due to the higher solubility of dodecanoic acid. However, the majority of undecanal produced was converted to 1‐undecanol. Although reduction of tridecanal to 1‐tridecanol was also detected, 1‐tridecanol was the minor product. Reduction of pentadecanal and heptadecanal to the corresponding alcohols was not detected. These results indicate that the endogenous alcohol dehydrogenases have a preference for shorter chain‐length aldehydes at pH 5.8 and deletion of endogenous alcohol dehydrogenases may be necessary for accumulation of undecanal and tridecanal. The highest aldehyde titers were achieved at pH 7.0. Production of undecanal, tridecanal, pentadecanal, and heptadecanal reached 406.2, 692.0, 2,332.9, and 280.7 μg/L, which were 35.0‐, 22.8‐, 63.8‐, and 212.7‐fold higher than those produced in unbuffered growth medium, respectively.

**Figure 2:**
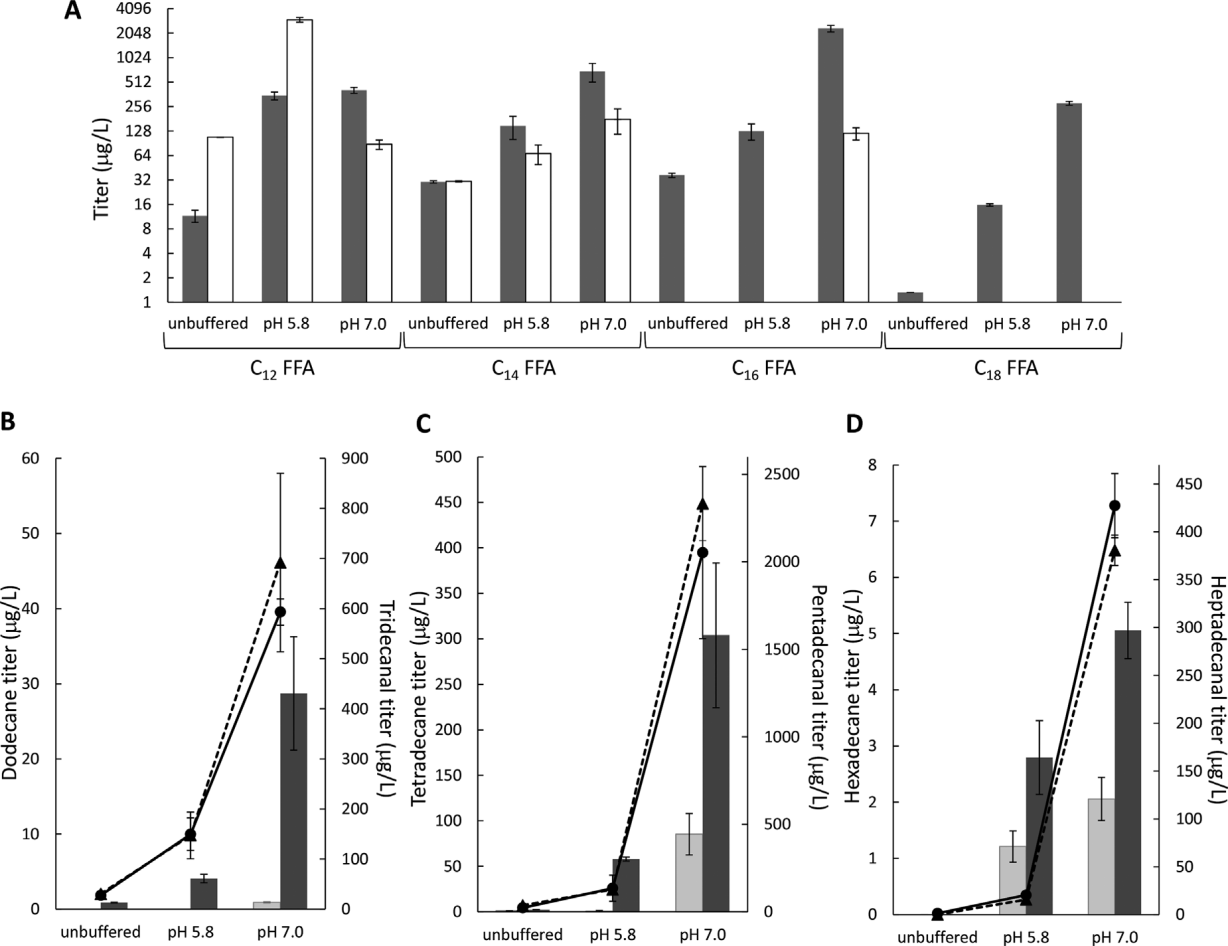
Whole‐cell biocatalytic conversion of exogenous FFAs to aldehydes and alkanes. JL1‐ctrl cultures at different pHs were fed with even chain‐length C_12‐18_ FFA and the titers for the C_n‐1_ aldehydes (gray bars) and the side products, C_n‐1_ alcohols (white bars), after 48 h of cultivation are shown in (A). For alkane production, JL1‐cADO cultures at different pHs were fed with the same FFAs as before. The titers of the corresponding C_n‐1_ aldehydes (circle, solid line) and C_n‐2_ alkanes (dark grey bar) produced from tetradecanoic, hexadecanoic and octadecanoic acids are illustrated in (B)‐(D), respectively. JL1‐ctrl cultures were cultivated in parallel, and the corresponding aldehyde (triangle, dotted line) and alkane (light grey bar) titers are shown (B)‐(D). No alkane was produced from the cultures fed with dodecanoic acid. The error bars show the standard deviation of biological duplicate.

For whole‐cell biocatalytic alkane production, JL1 was transformed with pYES2‐cADO to generate the strain JL1‐cADO for overexpression of cADO under the galactose‐inducible GAL1 promoter. Cultures of JL1‐cADO were similarly supplemented with even chain‐length C_12–18_ FFAs. Under unbuffered growth conditions, only miniscule amounts of alkane was produced (<3 μg/L) due to the low production of aldehydes. By raising the pH of the cultures with phosphate buffer, the increased aldehyde titers led to markedly enhanced alkane production (Fig. 2B–D). The titers for dodecane, tetradecane, and hexadecane reached 28.7, 304.0, and 5.1 μg/L at pH 7.0 but no decane was observed. No extracellular alkane was detected, possibly due to the low quantity of alkane produced (Buijs et al., 2014). Interestingly, even without expression of cADO, alkanes were detected in JL1‐ctrl upon production of aldehydes from the supplemented FFAs, although the alkane titers were significantly lower than JL1‐cADO. Since aldehydes are known to cause accumulation of reactive oxygen species, including free radicals (Allen et al., 2010), one possible reason for the alkanes observed in the absence of cADO could be non‐enzymatic free radical decarbonylation of aldehydes mediated by thiols (e.g., cysteine) (Barrett and Waters, 1953). Nevertheless, we have established that it is advantageous to cultivate the engineered strains at pH 7.0 for enhanced fatty acid utilization in our pathway for aldehyde and alkane production. Moreover, our results demonstrated the potential of our engineered *S. cerevisiae* possessing the alkane production pathway for whole‐cell biocatalytic conversion of exogenous FFA feedstocks to alkanes.

Building on our results from optimizing the alkane production pathway for whole‐cell biocatalysis, we proceeded to study the feasibility of engineering *S. cerevisiae* for de novo biosynthesis of alkane from simple sugar. We integrated P_TEF1_‐αDOX into the genome of *S. cerevisiae* BY4741 *faa1Δ faa4Δ*, an FFA‐overproducing strain generated by deleting the fatty acyl‐CoA synthethases *FAA1* and *FAA4* to prevent FFAs generated during lipid remodeling from being recycled (Scharnewski et al., 2008). The resulting strain, JL1FA, constitutively expressed αDOX for conversion of the accumulated FFAs into aldehydes, rendering it a self‐sufficient aldehyde‐producing strain. We attempted de novo production of alkane by transforming pYES2‐cADO into JL1FA to generate the strain JL1FA‐cADO. However, despite the ability of JL1FA‐cADO to produce 1,207.6 μg/L aldehyde at pH 7.0, no alkane was observed. We hypothesized that the continuous production of the relatively reactive aldehyde in JL1FA may have a deleterious effect on the cell physiology and aldehyde deformylating activity, for example, by forming reactive oxygen species and aldehyde–protein adducts (Allen et al., 2010; Setshedi et al., 2010). To overcome this obstacle, we hypothesized that if the cADO was constitutively expressed and the expression of αDOX was regulated, cADO would be functionally expressed first and might facilitate more efficient conversion of the aldehydes formed when the expression of αDOX was subsequently induced.

To test our hypothesis, αDOX and cADO were cloned into an expression plasmid in which αDOX was expressed under the galactose‐inducible GAL1 promoter while cADO was expressed constitutively from the TEF1 promoter (Supplementary Fig. S1). The resulting plasmid was transformed into BY4741 *faa1Δ faa4Δ* (resulting in strain JL2‐cADO), and the aldehyde and alkane productions were analyzed over 96 h in 24 h intervals. A control strain, JL2‐ctrl, with cADO replaced by an inactive mutant of cADO, was cultured in parallel. After 24 h of cultivation, tridecanal, pentadecanal, and heptadecanal were produced from the accumulated tetradecanoic, hexadecanoic, and octadecanoic acids (Fig. 3), respectively. At this time point, tetradecane and hexadecane (which were converted from pentadecanal and heptadecanal, respectively) were detected but the differences between the titers in the production strain JL2‐cADO and the control strain JL2‐ctrl were too small to conclude that cADO contributed to the alkane production. However, at the 48‐h time point, alkane production in JL2‐cADO (42.4 μg/L tetradecane; 31.1 μg/L hexadecane) was evidently higher than that in JL2‐ctrl (23.8 μg/L tetradecane; 18.1 μg/L hexadecane), hence validating the activity of cADO and demonstrating de novo alkane production in JL2‐cADO. This time point also corresponded to the peak of alkane and aldehyde titers. Beyond 48 h of cultivation, the cells entered stationary phase (Supplementary Fig. S2) and the aldehyde titers decreased while alkane titers plateaued. Interestingly, the titers of FFAs peaked only after culturing for 72 h, when there was 23.3, 124.5, 33.3 mg/L intracellular tetradecanoic, hexadecanoic and octadecanoic acid, respectively (Supplementary Fig. S3), thus indicating that the decrease in aldehyde titer was not due to insufficient FFA precursors. The inability of the strains to further biotransform the FFAs into aldehydes after 72 h of cultivation might be due to reduced αDOX activity and expression from the GAL1 promoter in the stationary phase (Lee and DaSilva, 2005). Moreover, no further increase in alkane titer was observed in the stationary phase although aldehydes were still available. No extracellular alkane was detected, thus, ruling out efflux of alkanes as the reason for the stagnated alkane titer and indicating that the activity of cADO was inhibited. The reason for the inhibited cADO activity is unclear although aging yeast cells are known to experience “redox collapse” (Brandes et al., 2013), which may prevent reversion of cADO to its active redox state (Paul et al., 2013). Additionally, it should be noted that no production of dodecane was observed, possibly due to the low titer of tridecanal. In order to increase production of tridecanal and shorter aldehydes, deletion of alcohol dehydrogenases may be beneficial for aldehyde accumulation by minimizing reduction of the aldehyde intermediates to alcohols. As cADOs are known to have low turnover number (Li et al., 2012), other enzymes, such as aldehyde decarbonylases from plants and insects (Bernard et al., 2012; Qiu et al., 2012), need to be explored to dramatically increase alkane titers.

**Figure 3:**
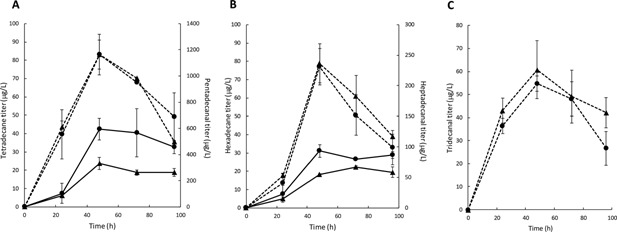
De novo biosynthesis of aldehydes and alkanes in FFA‐accumulating *S. cerevisiae*. JL2‐cADO (circle) and JL2‐ctrl (triangle) were cultivated at pH 7.0 and the aldehyde (dotted line) and alkane (solid line) titers were determined at 24, 48, 72, and 96 h time points. (A) shows the pentadecanal and tetradecane titers and (B) shows the heptadecanal and hexadecane titers. Although tridecanal was produced, as shown in (C), no dodecane was detected. The error bars show the standard deviation of biological duplicate.

In conclusion, we have demonstrated in this proof‐of‐principle study the engineering of *S. cerevisiae* for whole‐cell biocatalytic conversion of fatty acids into alkanes as well as de novo production of alkanes from simple sugar by optimizing the alkane production pathway and growth conditions. However, alkane production level in *S. cerevisiae* to date is still low, as shown in this work as well as those by Buijs et al. (2014) (22.0 μg/gDW of long chain C_13–17_ alkanes) and Bernard et al. (2012) (19 μg/gDW of C_29_ alkane). To further enhance alkane biosynthesis in *S. cerevisiae*, accumulation of aldehydes may be improved by deleting endogenous alcohol dehydrogenases and alternative enzymes to cADO need to be investigated for more efficient conversion of aldehydes to alkanes. Regulation of the expression of αDOX and cADO may need further optimization using other promoters to better utilize the accumulated FFAs and improve deformylation of the aldehydes to alkanes. Nonetheless, the engineered *S. cerevisiae* strains described here are highly promising platforms for alkane production from FFAs and the findings in this work will facilitate future development of alkane‐producing yeast hosts.

## Materials and Methods

### Strains, Oligonucleotides, Chemicals, and Culture Media

The yeast strains used in this study were generated from *S. cerevisiae* BY4741 (ATCC). Molecular biology reagents were purchased from New England Biolabs (Beverly, MA). Oligonucleotides were synthesized by Integrated DNA Technologies (Singapore). The genes for αDOX (NCBI Gene ID: 4352160) and cADO from *Synechococcus elongatus* PCC 7942 (NCBI Gene ID: 3775017) were codon‐optimized for yeast expression and synthesized by Genscript (Nanjing, China). pYES2/CT plasmid was purchased from Thermo Fisher (Waltham, MA). Yeast extract, peptone, and tryptone were procured from BD Biosciences (San Jose, CA). All other chemicals were purchased from Sigma Aldrich (St. Louis, MO) unless otherwise stated. All plasmids, yeast strains, and oligonucleotides are listed in Supplementary Tables I, II, and III, respectively. Details on plasmid construction are available in Supplementary Information.

YPD medium (1% yeast extract, 2% peptone, and 2% dextrose) was used for non‐selective cultivation of yeast strains. Yeast transformants with URA3 selection marker were cultivated in yeast minimal medium consisting of yeast nitrogen base (YNB, 6.7 g/L) supplemented with 1.92 g/L uracil‐deficient amino acid dropout mixture, 50 μM FeCl_3_ and 2% dextrose, 2% galactose or 0.2% glucose with 1.8% galactose (hereafter referred as YD‐U, YG‐U, and YDG‐U, respectively). Solid growth media were similarly prepared with addition of 2% agar to the recipe described.

### Construction of Yeast Strains

Yeast competent cells were prepared and DNAs were transformed using the LiOAc/PEG method (Gietz and Schiestl, 2007). BY4741 *faa1Δ faa4Δ* was generated as described in literature (Chen et al., 2015). JL1 and JL1FA strains were constructed by transforming BY4741 and BY4741 *faa1Δ faa4Δ*, respectively, with NruI‐digested pIS385‐DOX to integrate P_TEF1_‐DOX‐T_ADH1_ into the LYS2 locus. The transformants were selected and the URA3 selection marker was removed as described in literature (Sadowski et al., 2007) to generate JL1 and JL1FA. JL1 was transformed with pYES2/CT and pYES2‐cADO to create JL1‐ctrl and JL1‐cADO strains. Similarly, JL1FA was transformed with pYES2/CT and pYES2‐cADO to create JL1FA‐ctrl and JL1FA‐cADO strains. JL2‐ctrl and JL2‐cADO were generated by transforming BY4741 *faa1Δ faa4Δ* with pUdGT‐DOX‐xADC and pUdGT‐DOX‐cADO.

### Cultivation of Strains for Production of Aldehydes and Alkanes

Cells from 5‐mL YD‐U overnight starter cultures of yeast strains were washed with sterile deionized water and diluted to OD_600_ ∼ 0.4 in 25 mL of YG‐U (for JL1‐ctrl, JL1‐cADO, JL1FA‐ctrl, and JL1FA‐cADO) or YDG‐U (for JL2‐ctrl and JL2‐cADO). The cultures were cultivated at 30°C and 225 rpm. When JL1‐ctrl, JL1‐cADO, JL1FA‐ctrl, and JL1FA‐cADO were used for biocatalytic conversion of exogenous fatty acids, the growth media was unbuffered or supplemented with 50 mM sodium phosphate buffer (pH 5.8 or 7.0). After 18 h of growth, 0.5 mL of even chain‐length C_12–18_ fatty acid solutions (10 mg/L in isopropanol) were added to the cultures. The cells were cultivated for an additional 48 h and harvested by centrifugation (3 min, 4,000*g*). For de novo production of aldehydes and alkanes using JL2‐ctrl and JL2‐cADO, the cells were cultured in medium supplemented with 50 mM sodium phosphate buffer (pH 7.0). The cell cultures were harvested by centrifugation (3 min, 4,000*g*) after 24, 48, 72, and 96 h of cultivation. All culture conditions were performed in biological duplicates.

### Analysis of Alcohols, Aldehydes, and Alkanes

To extract alcohols, aldehydes, and alkanes, the cell pellets were resuspended in 400 μL deionized water, and 5 μL 1‐octadecene (1 mg/mL) was added as internal standard. Subsequently, 500 μL ethyl acetate was added, and the cells were disrupted by bead beating. The ethyl acetate extracts were analyzed by gas–liquid chromatography (GC) using an Agilent 7890B GC system equipped with an HP‐5MS column (Agilent) coupled to a mass spectrometer (MS, Agilent 5977). The program used for GC analysis was as follow: 40°C for 0.5 min followed by ramping up to 280°C at a rate of 10°C/min and a final hold at 280°C for 4 min. Quantification was performed with calibration plots obtained using alcohol, aldehyde (purchased from TCI, Japan), and alkane standards. Fatty acid analysis is described in Supplementary Information.

This work was supported by the Competitive Research Program of the National Research Foundation of Singapore (NRF‐CRP5‐2009‐03) and the Synthetic Biology Initiative of the National University of Singapore (DPRT/943/09/14).

## Supporting information

Additional supporting information may be found in the online version of this article at the publisher's web‐site.

Table S1. Plasmids used in this study.
Table S2. EStrains used in this study.
Table S3. Oligonucleotides used in this study.
Figure S1. Plasmid map of pUdGT‐DOX‐cADO and pUdGT‐DOX‐xADO.
Figure S2. Growth profiles of JL2‐ctrl and JL2‐cADO. The growth curves for JL2‐ctrl and JL2‐cADO are shown in black and blue, respectively. Error bars show standard deviation of biological duplicate.
Figure S3. Time‐course study of fatty acid accumulation in JL2‐cADO. Tetradecanoic (A), hexadecanoic (B), and octadecanoic (C) acids of JL2‐cADO cultures were quantified at 24, 48, 72, and 96 h time points. Extracellular and intracellular fatty acids are shown as grey and blue bars, respectively. Error bars show standard deviation of biological duplicate.
