# Supplementary material for: Whole‐cell biocatalytic and de novo production of alkanes from free fatty acids in Saccharomyces cerevisiae
Source: Biotechnol Bioeng. 2016 Jan 19;114(1):232–7. doi: 10.1002/bit.25920 (PMC5132040; doi:10.1002/bit.25920)
Supplement: Supplementary file 1 — Table S1. Plasmids used in this study. Table S2. EStrains used in this study. Table S3. Oligonucleotides used in this study. Figure S1. Plasmid map of pUdGT‐DOX‐cADO and pUdGT‐DOX‐xADO. Figure S2. Growth profiles of JL2‐ctrl and JL2‐cADO. The growth curves for JL2‐ctrl and JL2‐cADO are shown in black and blue, respectively. Error bars show standard deviation of biological duplicate. Figure S3. Time‐course study of fatty acid accumulation in JL2‐cADO. Tetradecanoic (A), hexadecanoic (B), and octadecanoic (C) acids of JL2‐cADO cultures were quantified at 24, 48, 72, and 96 h time points. Extracellular and intracellular fatty acids are shown as grey and blue bars, respectively. Error bars show standard deviation of biological duplicate. [file BIT-114-232-s001.docx]

**SUPPLEMENTARY INFORMATION**

**CONTENT Page No.**

Supplementary Table I: Plasmids used in this study ………............................................ 2

Supplementary Table II: Strains used in this study …........................................................ 2

Supplementary Table III: Oligonucleotides used in this study ........................................ 2

Supplementary Figure S1: Plasmid map of pUdGT-DOX-cADO and pUdGT-DOX-xADO ............................................................................................................................ 3

Supplementary Figure S2: Growth profiles of JL2-ctrl and JL2-cADO…....................... 4

Supplementary Figure S3. Time-course study of fatty acid accumulation in JL2-cADO... 5

Supplementary Methods: Plasmid construction................................................................. 6

Supplementary Methods: Analysis of fatty acids………................................................... 7

References………........………........………........………................................................... 8

**Supplementary Table I.** Plasmids used in this study

| **Strains** | **Description** | **Source** |
| --- | --- | --- |
| pESC-URA |  | Agilent |
| pYES2/CT |  | Thermo Fisher |
| pYES2-cADO | pYES2/CT with P_GAL1_-ADC | This study |
| pGT | pESC-URA with P_GAL10_-P_GAL1_ replaced with P_GAL1_-P_TEF1_ | This study |
| pUdGT | pESC-GT with truncated URA3 promoter | This study |
| pUdGT-DOX | pESC-UdGT with P_GAL1_-DOX | This study |
| pUdGT-DOX-cADO | pESC-UdGT-DOX with P_TEF1_-ADC | This study |
| pUdGT-DOX-xADO | pESC-UdGT-DOX with P_TEF1_-ADC | This study |
| pIS385-DOX | pIS385 carrying P_TEF1_-DOX-T_ADH1_ | This study |

**Supplementary Table II.** Strains used in this study

| **Strains** | **Description** | **Source** |
| --- | --- | --- |
| BY4741 | MATa *his3Δ1 leu2Δ0 met15Δ0 ura3Δ0* | ATCC |
| BY4741 *faa1Δ faa4Δ* | MATa *his3Δ1 leu2Δ0 met15Δ0 ura3Δ0 faa1Δ faa4Δ* | (Chen et al. 2015) |
| JL1 | BY4741, *lys2*::P_TEF1_-DOX -T_ADH1_ | This study |
| JL1-ctrl | JL1 carrying pYES2/CT | This study |
| JL1-cADO | JL1 carrying pYES2-cADO | This study |
| JL1FA | BY4741 *faa1Δ faa4Δ*, *lys2*::P_TEF1_-DOX -T_ADH1_ | This study |
| JL1FA-ctrl | JL1FA carrying pYES2/CT | This study |
| JL1FA-cADO | JL1FA carrying pYES2-cADO | This study |
| JL2-ctrl | BY4741 *faa1Δ faa4Δ* carrying pUdGT-DOX-xADO | This study |
| JL2-cADO | BY4741 *faa1Δ faa4Δ* carrying pUdGT-DOX-cADO | This study |

**Supplementary Table III.** Oligonucleotides used in this study

| **Name** | **Sequences** |
| --- | --- |
| pIS385-DOX-F | TAGCAATCTAATCTAAGTTTTAATTACAAAAATGGGTTCTGGTTTGTTTAAG |
| pIS385-DOX-R | TCATAAATCATAAGAAATTCGCTTATTTAGTTAATGGTGATGGTGATGGTGGTAG |
| pIS385-TEF1p-F | TTCTATGAGCTCTTTCATAGCTTCAAAATGTTTCTAC |
| pIS385-TEF1p-R | TTTGTAATTAAAACTTAGATTAGAT |
| pIS385-ADH1t-F | CTAAATAAGCGAATTTCTTATGATT |
| pIS385-ADH1t-R | TGTAGAGGTACCTTTCAGCTGAATTGGAGCGACCTCATGC |
| cADO-F | TGTAGAGGATCCAAAAATGCCTCAATTGGAAGCTTCT |
| cADO-R | GCCAGTCTCGAGTTAATGGTGATGGTGATGGTGAACAGCAGCCAAACCATAAGCAGAC |
| pESC-pmt-GAL1-F | AGTACGGATTAGAAGCCGCCGAGCG |
| pESC-pmt-GAL1-R | GCCAGTGAATTCGAATGGGTTTTTTCTCCTTGACGTTAAAG |
| pESC-pmt-TEF1-F | CGCTCGGCGGCTTCTAATCCGTACTTTTCATAGCTTCAAAATGTTTCTAC |
| pESC-pmt-TEF1-R | TATTACGGATCCGTTTGTAATTAAAACTTAGATTAGAT |
| P17-URA3-F | TTTCATGTGCACTTTTTTTAGGAAACGAAGATAAATCATGTCG |
| P17-URA3-R | TTCTGTCTTCGAAGAGTAAAAAATT |


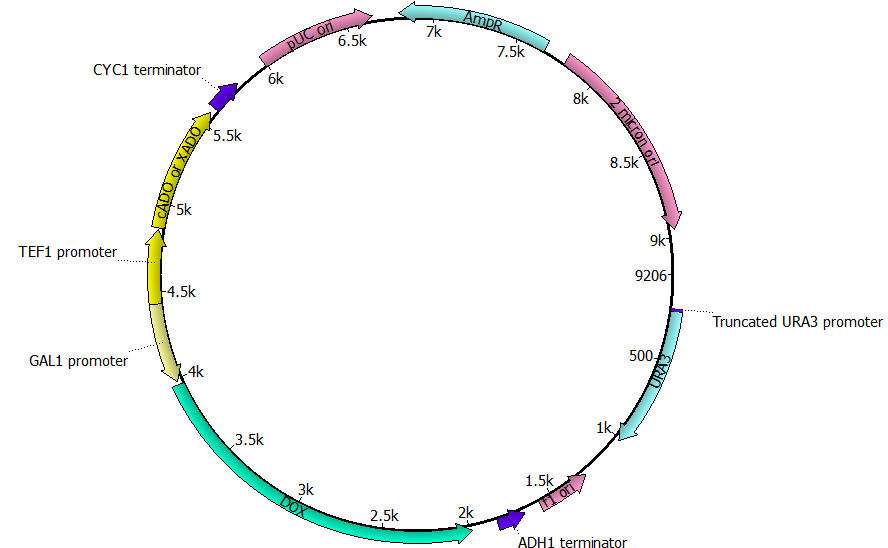


**Supplementary Figure S1.** Plasmid map of pUdGT-DOX-cADO and pUdGT-DOX-xADO

**
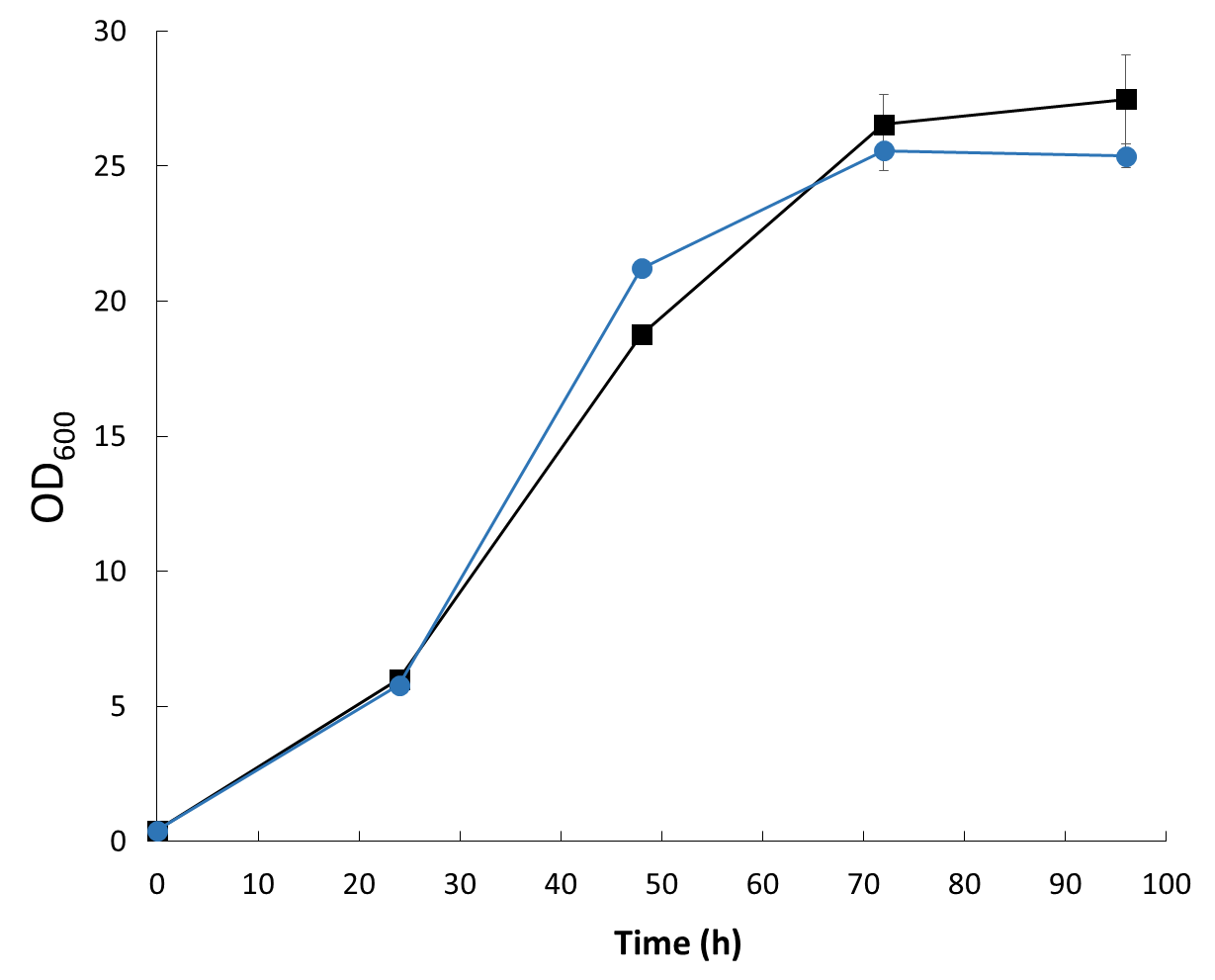
Supplementary Figure S2.** Growth profiles of JL2-ctrl and JL2-cADO. The growth curves for JL2-ctrl and JL2-cADO are shown in black and blue, respectively. Error bars show standard deviation of biological duplicate.

**
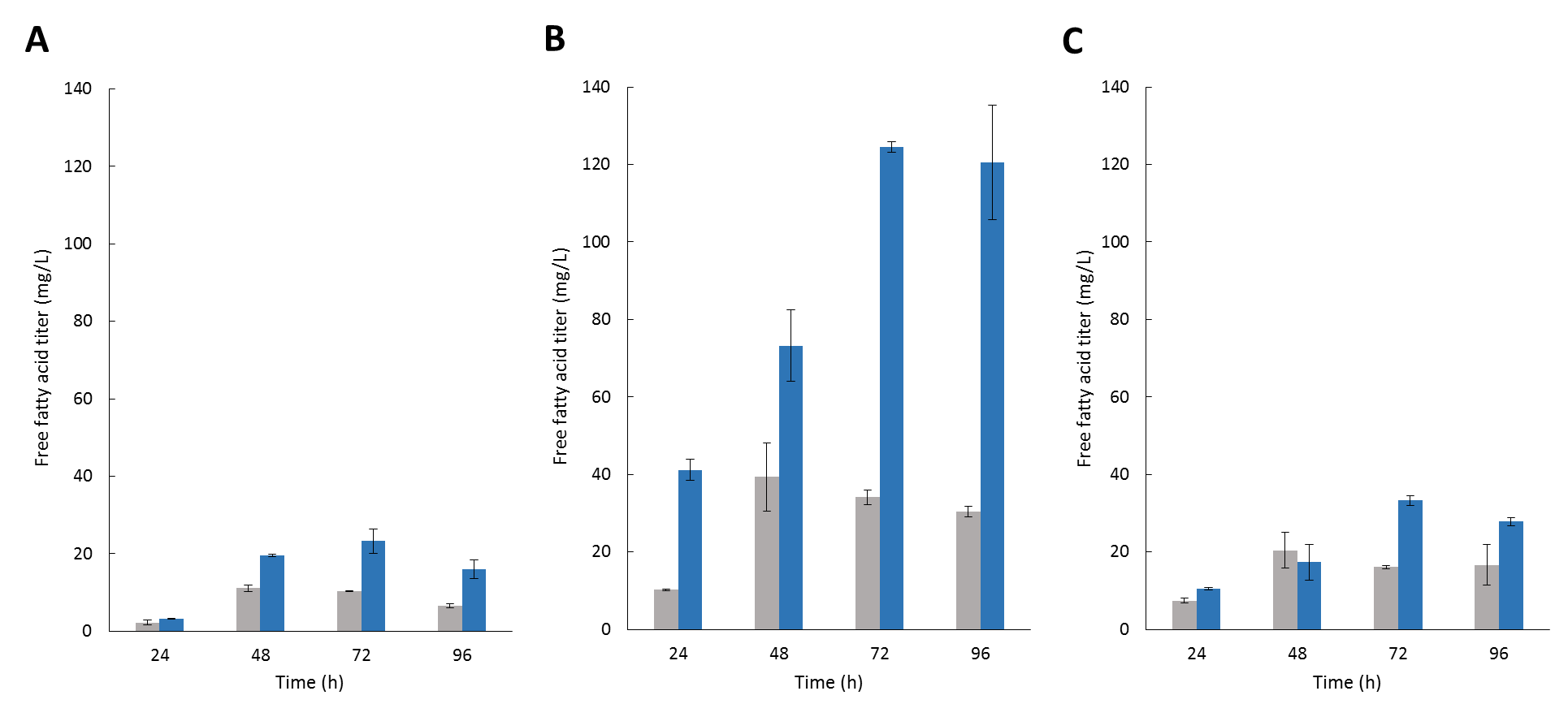
**

**Supplementary Figure S3.** Time-course study of fatty acid accumulation in JL2-cADO. Tetradecanoic (A), hexadecanoic (B) and octadecanoic (C) acids of JL2-cADO cultures were quantified at 24, 48, 72 and 96 h time points. Extracellular and intracellular fatty acids are shown as grey and blue bars, respectively. Error bars show standard deviation of biological duplicate.

**Supplementary Methods**

**Plasmid construction**

Episomal plasmids used for protein expression in yeast were pYES2/CT (Thermo Fisher) and pESC-URA (Agilent). pIS385 was obtained from Euroscarf for integrating genes into the genome at the LYS2 locus (Sadowski et al. 2007). The genes for αDOX (NCBI Gene ID: 4352160), cADO (NCBI Gene ID: 3775017) were codon-optimized for yeast expression. The Kozak sequence AAAA was added before the start codon of DOX and cADO, and the sequences were synthesized and provided as plasmids pUG57-DOX and pUG57-cADO, respectively, by Genscript (China). Plasmids constructed in this study were propagated using *E. coli* TOP10 (Invitrogen). *E. coli* was grown in lysogeny broth (1% tryptone, 0.5% yeast extract and 1% NaCl) supplemented with ampicillin (100mg/L). Solid growth media were similarly prepared with addition of 2% agar to the recipe described. Plasmids were isolated using QIAprep Spin Miniprep Kit (Qiagen). PCR purification and DNA gel extraction were performed with Wizard SV Gel and PCR Clean-Up System (Promega). All genes were sequenced to verify successful cloning.

*Plasmid pYES2-cADO*: cADO was digested from pUG57-cADO with HindIII/XhoI and cloned into pYES2/CT to obtain pYES2-cADO.

*Plasmid pGT*: pGT was constructed by replacing the P_GAL1_ and P_GAL10_ promoters of pESC-URA with P_TEF1_ and P_GAL1_ promoters, respectively. P_TEF1_ was amplified from the purified genomic DNA of S. cerevisiae strain BY4741 using the primer pairs pESC-pmt-TEF1-F/pESC-pmt-TEF1-R. P_GAL1_ was amplified from pESC-URA using the primer pairs pESC-pmt-GAL1-F/pESC-pmt-GAL1-R. The amplified promoter fragments were spliced by overlap extension PCR using primers pESC-pmt-TEF1-R and pESC-pmt-GAL1-R to generate the divergent P_TEF1_-P_GAL1_ promoter cassette. Subsequently, the promoter cassette was digested with EcoRI/BamHI before cloning into pESC-URA to replace the original P_GAL10_/P_GAL1_ segment.

*Plasmid pUdGT*: pUdGT was constructed by truncating the promoter sequence of the URA3 selection marker on pGT for increased plasmid stability (Seresht et al. 2013). pESC-URA was digested with XbaI/ApaLI to recover a 615-bp fragment (F1) by gel extraction. A DNA fragment containing the last 17-bp of the URA3 promoter was amplified using the primer pair P17-URA3-F/P17-URA3-R and digested with ApaLI/EcoRV to generate fragment F2. pGT was digested with XbaI/EcoRV and the 5.7kb fragment was ligated with F1 and F2 to obtain pUdGT.

*Plasmid pUdGT-DOX*: DOX was digested from pUG57-DOX with NotI/SacI and cloned into the corresponding restriction site in pUdGT to obtain pUdGT-DOX.

*Plasmid pUdGT-DOX-cADO*: pUdGT-DOX-cADO was created by amplifying cADO from pUG57-cADO with cADO-F/cADO-R, digesting the amplicon with BamHI/XhoI and cloning the DNA fragment into pUdGT-DOX.

*Plasmid pUdGT-DOX-xADO*: E60 and H63 are the metal binding residues of cADO based on literature (Jia et al. 2015) and crystal structure (PDB ID: 4QUW). Therefore, cADO was inactivated by mutating both E60 and H63 to alanine to create xADO. Mutagenesis was performed by amplify cADO from pUG57-cADO with the primer pairs cADO-F/xADO-R and xADO-F/cADO-R. The two fragments were spliced by overlap extension PCR using cADO-F/cADO-R. The resulting amplicon was digested with BamHI/XhoI and cloned into pUdGT-DOX to created pUdGT-DOX-xADO.

*Plasmid pIS385-DOX*: P_TEF1_ was amplified using the primers pIS385-TEF1p-F/pIS385-TEF1p-R from the genomic DNA of BY4741. T_ADH1_ was amplified using the primers pIS385-ADH1t-F/pIS385-ADH1t-R. DOX gene fragment was amplified from pUG57-DOX using primers pIS385-DOX-F/pIS385-DOX-R. The three DNA fragments were assembled by overlap extension PCR using the primers pIS385-TEF1p-F/pIS385-ADH1t-R to obtain the P_TEF1_-DOX-T_ADH1_ insert cassette. P_TEF1_-DOX-T_ADH1_ was digested with KpnI/SacI and cloned into pIS385 (Sadowski et al. 2007) to obtain pIS385-DOX.

**Analysis of fatty acids**

To extract total fatty acids, 500 µL ethyl acetate was added to 500 µL of culture and the cells were disrupted by bead beating. To extract extracellular fatty acids, 500 µL of culture was centrifuged (4000 g, 3 min) and the supernatant was vortexed for 1 min with 500 µL ethyl acetate. 1-Octadecene was used as internal standard. The ethyl acetate extracts were derivatized with N,O-bis(trimethylsilyl)trifluoroacetamide and the samples were analyzed by gas-liquid chromatography (GC) using an Agilent 7890B GC system equipped with an HP-5MS column (Agilent) coupled to a mass spectrometer (MS, Agilent 5977). The program used for GC analysis was as follow: 40°C for 0.5 min followed by ramping up to 280°C at a rate of 10°C/min and a final hold at 280°C for 4 min. Quantification was performed with calibration plots obtained using fatty acid standards.

**References**

Chen B, Lee DY, Chang MW. 2015. Combinatorial metabolic engineering of Saccharomyces cerevisiae for terminal alkene production. Metab Eng 31:53-61.

Jia CJ, Li M, Li JJ, Zhang JJ, Zhang HM, Cao P, Pan XW, Lu XF, Chang WR. 2015. Structural insights into the catalytic mechanism of aldehyde-deformylating oxygenases. Protein & Cell 6(1):55-67.

Sadowski I, Su TC, Parent J. 2007. Disintegrator vectors for single-copy yeast chromosomal integration. Yeast 24(5):447-455.

Seresht AK, Norgaard P, Palmqvist EA, Andersen AS, Olsson L. 2013. Modulating heterologous protein production in yeast: the applicability of truncated auxotrophic markers. Applied Microbiology and Biotechnology 97(9):3939-3948.
